# Supplementary figures and images for: Novel, non-symbiotic isolates of Neorhizobium from a dryland agricultural soil
Source: PeerJ. 2018 May 16;6:e4776. doi: 10.7717/peerj.4776 (PMC5960266; doi:10.7717/peerj.4776)

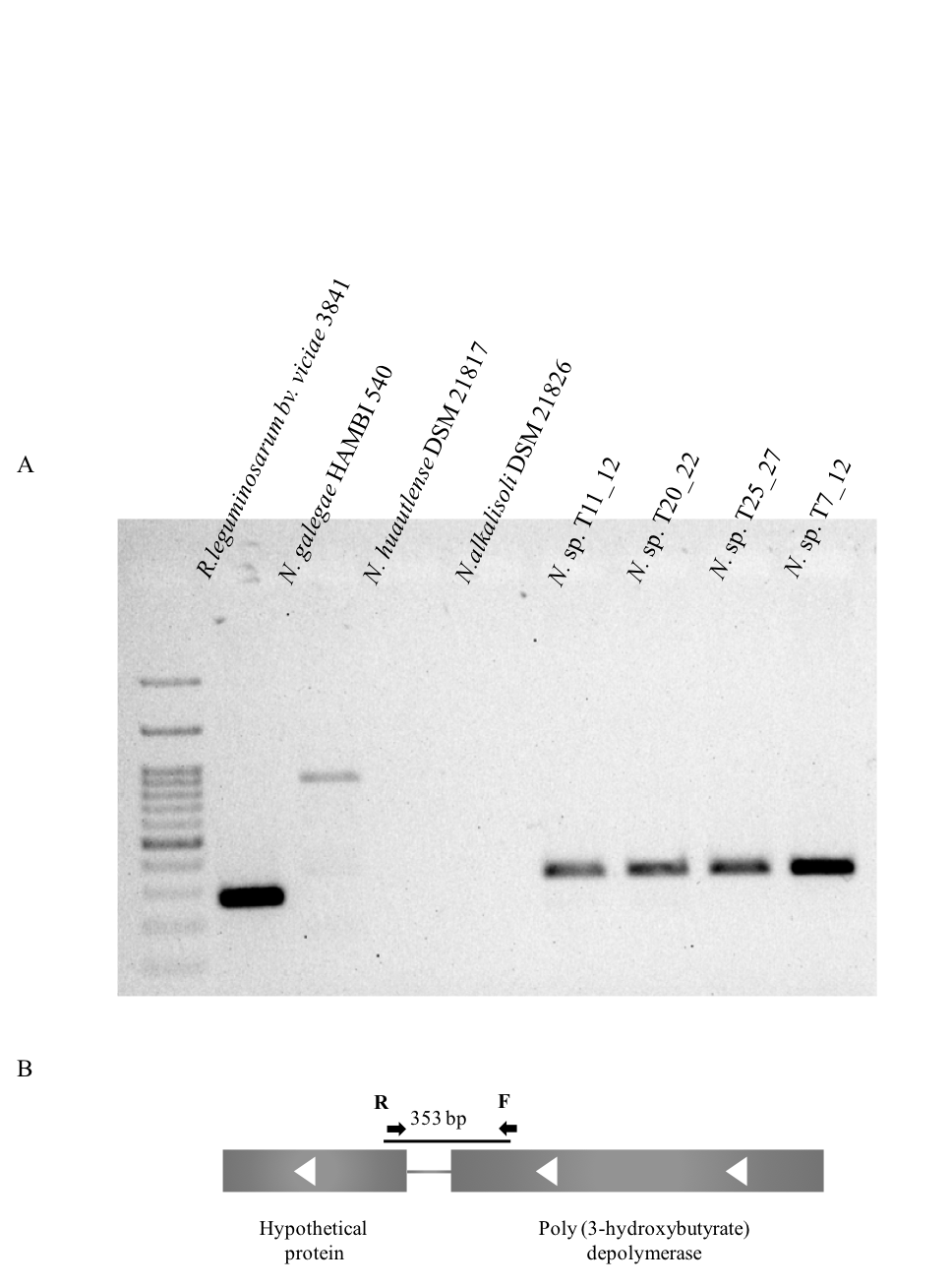

Supplement: Figure S1 — (A) Agarose gel electrophoresis separation of PCR products. (B) Schematic representation of the genomic region amplified by fnrN primers in Tomejil Neorhizobium sp. strains (see text). Genomic sequences partially complementary to fnrN primers were identical in all Tomejil strains: F_fnrN (5′-GGTGATGCCGGACGGGCG-3′) hybridized to 5′-ccgctcGCCGGtCGGGCG -3′, and R_fnrN (5′- TGGAGCAGGCTTGCGACCTT-3′) hybridized to 5′-cGGAtCAGGtTTGCGACCTT-3′. [file peerj-06-4776-s001.png]
