# Supplementary material for: Novel, non-symbiotic isolates of Neorhizobium from a dryland agricultural soil
Source: PeerJ. 2018 May 16;6:e4776. doi: 10.7717/peerj.4776 (PMC5960266; doi:10.7717/peerj.4776)
Supplement: Figure S2 — Multiple sequence alignment of partial rpoB sequences corresponding to PCR amplified fragments of the 20 Tomejil rpoB sequence groups (see legend to Fig. 2), and equivalent sequences from type strains of the known Neorhizobium species. [file peerj-06-4776-s002.pdf]

```
CLUSTAL O(1.2.4) multiple sequence alignment


A.tumefaciens      CGTGACCCGCTCCTGATGGCGCTCGGCATGGACGGCGAAGAAATCCTGTCGACCTTCTAT  60
N.galegae          CGTGACCCGCTGCTGATGGCGCTTGGCATGGACGGCGAAGAAATCCTGTCGACCTTCTAT  60
T4_1               GTGACCTCGCTGCTGATGGCGCTCGGCATGGACGGCGAAGAAATCCTGTCGACCTTCTAT  60
T4_8               GTGACCTCGCTTCTGATGGCGCTTGGCATGGACGGCGAAGAAATCCTGTCGACCTTCTAT  60
T22_47             GTGACCTCGCTGCTGATGGCGCTCGGCATGGACGGCGAAGAAATCCTGCCCaCCTTCTAT  60
T7_7               GTGACCTCGCTGCTGATGGCGCTCGGCATGGACGGCGAAGAAATCCTGTCGACCTTCTAC  60
T5_2               GTGACCTCGCTGCTGATGGCGCTCGGCATGGACGGCGAAGAAATCCTGTCGACCTTCTAT  60
T7_11              GTGACCTCGCTGCTGATGGCGCTCGGCATGGACGGCGAAGAAATCCTGTCGACCTTCTAT  60
T6_21              GTGACCTCGCTGCTGATGGCGCTCGGCATGGACGGCGAAGAAATCCTGTCGACCTTCTAT  60
T7_12              GTGACCTCGCTGCTGATGGCGCTCGGCATGGACGGCGAAGAAATCCTGTCGACCTTCTAT  60
T16_9              GTGACCTCGCTGCTGATGGCGCTCGGCATGGACGGCGAAGAAATCCTGCCCACCTACAAT  60
T5_26              GTGACCTCGCTCTTGATGGCGCTCGGCATGGACGGCGAAGAAATCCTGTCGACCTTCTAT  60
T24_19             GTGACCTCGCTGCTGATGGCGCTCGGCATGGACGGCGAAGAAATCCTGTCGATCTTCACT  60
T20_10             GTGACCTCGCTGCTGATGGCACTCGGCATGGACGGCGAAGAAATCCTGTCGACCTTCTAT  60
T11_12             GTGACCTCGCTGCTGATGGCGCTCGGCATGGACGGCGAAGAAATCCTGTCGACCTTCTAT  60
T21_15             GTGACCTCGCTGCTGATGGCGCTCGGCATGGACGGCGAAGAAATCCTGTCGACCTTCTAC  60
T25_7              GTGACCTCGCTGCTGATGGCGCTTGGCATGGATGGCGAAGAAATCCTGTCGACCTTCTAC  60
T6_1               GTGACCTCGCTGCTGATGGCGCTCGGCATGGACGGCGAAGAAATCCTGTCGACCTTCTAC  60
T13_2              GTGACCTCGCTGCTGATGGCGCTCGGCATGGACGGCGAAGAAATCCTGTCGACCTTCTAT  60
T25_13             GTGACCTCGCTGCTGATGGCGCTCGGCATGGACGGCGAAGAAATCCTGTCGACCTTCTAC  60
T23_12             GTGACCTCGCTGCTGATGGCGCTCGGCATGGACGGCGAAGAAATCCTGTCGACCTTCTAT  60
T6_25              GTGACCTCGCTGCTGATGGCGCTCGGCATGGACGGCGAAGAAATCCTGTCGACCTTCTAT  60
N.alkalisoli       GGTATCGCGCTGCTGATGGCGCTTGGAATGGATGGCGAAGACATTCTGTCGACCTTCTAT  60
N.huautlense       GGTATCTCGCTGCTGATGGCGCTCGGGATGGACGGCGAAGACATTCTGTCGACCTTCTAT  60
                        * ****  ******* ** ** ***** ******** ** *** * * ** *

A.tumefaciens      ACCAAGGCTACCTACGAGCGCGCCGGTGACGGCTGGCGTATCCCGTTCCAGCCTGACGCG  120
N.galegae          ACGAAGTCGTCCTATCAGCGCGACGGCGATGGCTGGCGGATTCCGTTCCAGCCGGAAACT  120
T4_1               ACGAAGTCGTCCTACCAGCGCGACGGCGAAGGCTGGCGGATTCCGTTCCAGCCGGAAACC  120
T4_8               ACGAAGTCGTCCTACCAGCGCGACGGCGAAGGCTGGCGGATTCCGTTCCAGCCGGAAACC  120
T22_47             ACGAAGTCGTCCTATCAGCGCGACGGCGACGGCTCCCGGATTCCGTTCCAGCCGGAAACC  120
T7_7               ACGAAGTCGTCCTACCAGCGCGACGGCGAAGGCTGGCGGATTCCGTTCCAGCCGGAAACC  120
T5_2               ACGAAGTCGTCCTATCAGCGCGACGGCGAAGGCTGGCGGATTCCGTTCCAGCCGGAAACC  120
T7_11              ACGAAGTCGTCCTATCAGCGCGACGGCGACGGCTGGCGGATTCCGTTCCAGCCGGAAACC  120
T6_21              ACGAAGTCGTCCTATCAGCGCGACGGCGACGGCTGGCGGATTCCGTTCCAGCCGGAAACC  120
T7_12              ACGAAGTCGTCCTATCAGCGCGACGGCGACGGCTGGCGGATTCCGTTCCAGCCGGAAACC  120
T16_9              ACGAAGTCGTCCTATCAGCGCGACGGCGAAGGCTGCCGGATTCCGTTCCAGCCGGAAACC  120
T5_26              ACGAAGTCGTCCTATCAGCGCGACGGCGACGGCTGGCGGATTCCGTTCCAGCCGGAAACC  120
T24_19             ACGAAGTCGTCCTATCAGCGCGACGGCGACGGCTGGCGGATTCCGTTCCAGCCGGAAACC  120
T20_10             ACGAAGTCGTCCTATCAGCGCGACGGCGACGGCTGGCGGATTCCGTTCCAGCCGGAAACC  120
T11_12             ACGAAGTCGTCCTATCAGCGCGACGGCGACGGCTGGCGGATTCCGTTCCAGCCGGAAACC  120
T21_15             ACGAAGTCGTCCTATCAGCGCGACGGCGACGGCTGGCGGATTCCGTTCCAGCCGGAAACC  120
T25_7              ACGAAGTCGTCCTACCAGCGCGACGGCGAAGGCTGGCGGATTCCGTTCCAGCCGGAAACC  120
T6_1               ACGAAGTCGTCCTACCGGCGCGACGGCGAAGGCTGGCGGATTCCGTTCCAGCCGGAAACC  120
T13_2              ACGAAGTCGTCCTATCAGCGCGACGGCGAAGGCTGGCGGATTCCGTTCCAGCCGGAAACC  120
T25_13             ACGAAGTCGTCCTACCAGCGCGACGGCGAAGGCTGGCGGATTCCGTTCCAGCCGGAAACC  120
T23_12             ACGAAGTCGTCCTACCAGCGCGACGGCGAAGGCTGGCGGATTCCGTTCCAGCCGGAAACC  120
T6_25              ACGAAGTCGTCCTACCAGCGCGACGGCGAAGGCTGGCGGATTCCGTTCCAGCCGGAAACC  120
N.alkalisoli       TCGAAGTCCAACTACGAGCGCGATGGCGACGGCTGGCGGATTCCGTTCCAGCCGGAGACC  120
N.huautlense       TCCAAGTCCAACTACGAGCGCGATGGCGATGGCTGGCGGATTCCGTTCCAGCCGGAAACC  120
                    * *** *   ***   *****  ** ** ****  ** ** *********** **  *

A.tumefaciens      CTGAAGAATGCCAAGGTCATCACCGACATGATCGACGCCGACACCGGCGAAGTTGTTGTC  180
N.galegae          CTGAAGGGTGCCAAGACCCTTTCCGACATGATTGACGCCGATACCGGCGAAGTGGTTGTC  180
T4_1               CTGAAGGGTGCAAAAACCCTTTCCGACATGATCGACGCCGATACCGGCGAAGTGGTTGTC  180
T4_8               CTGAAGGGTGCAAAAACCCTTTCCGACATGATCGACGCCGATACCGGCGAAGTGGTTGTC  180
T22_47             CTGAAAGGTGCGAAGACCCTTTCCGACATGATCGACGCCGATACCGGCGAAGTGGTTGTC  180
T7_7               CTGAAAGGTGCGAAGACCCTTTCCGATATGATCGACGCCGATACCGGCGAAGTGGTTGTC  180
T5_2               CTGAAAGGTGCGAAGACCCTTTCCGATATGATCGACGCCGATACCGGCGAAGTGGTTGTC  180
T7_11              CTGAAGGGTGCGAAGACCCTTTCCGACATGATCGACGCCGATACCGGCGAAGTGGTTGTC  180
T6_21              CTGAAAGGTGCGAAGACCCTTTCCGACATGATCGACGCCGATACCGGCGAAGTGGTTGTC  180
T7_12              CTGAAAGGTGCGAAGACCCTTTCCGACATGATCGACGCCGATACCGGCGAAGTGGTTGTC  180
T16_9              CTGAAGGGTGCGAAGACCCTTTCCGACATGATCGACGCCGATACCGGCGAAGTGGTTGTC  180
T5_26              CTGAAGGGTGCGAAGACCCTTTCCGACATGATCGACGCCGATACCGGCGAAGTGGTTGTC  180
T24_19             CTGAAGGGTGCGAAGACCCTTTCCGACATGATCGACGCCGATACCGGCGAAGTGGTTGTC  180
T20_10             CTGAAGGGTGCGAAGACCCTTTCCGACATGATCGACGCCGATACCGGCGAAGTGGTTGTC  180
T11_12             CTGAAGGGTGCGAAGACCCTTTCCGACATGATCGACGCCGATACCGGCGAAGTGGTTGTC  180
T21_15             CTGAAGGGTGCGAAGACCCTTTCCGACATGATCGACGCCGATACCGGCGAAGTGGTTGTC  180
T25_7              CTGAAGGGTGCGAAGACCCTTTCCGACATGATCGACGCCGATACCGGCGAAGTGGTTGTC  180
T6_1               CTGAAGGGTGCGAAGACCCTTTCCGACATGATCGACGCCGATACCGGCGAAGTGGTTGTC  180
T13_2              CTGAAGGGTGCGAAGACCCTTTCCGACATGATCGACGCCGATACCGGCGAAGTGGTTGTC  180
T25_13             CTGAAGGGTGCGAAGACCCTTTCCGACATGATCGACGCCGATACCGGCGAAGTGGTTGTC  180
T23_12             CTGAAGGGTGCGAAGACCCTTTCCGACATGATCGACGCCGATACCGGCGAAGTGGTTGTC  180
T6_25              CTGAAGGGTGCGAAGACCCTTTCCGACATGATCGACGCCGATACCGGCGAAGTGGTTGTC  180
```

```
N.alkalisoli       TTGAAGGGCGCCAAGACTCTTTCAGACATGATCGACGCCGACACCGGCGAAGTGGTGGTC  180
N.huautlense       CTCAAGGGTGCCAAGACCCTTTCCGACATGATCGACGCCGACACCGGCGAAGTGGTCGTC  180
                    * **    ** **     *  * ** ***** ******** *********** ** ***

A.tumefaciens      GAGGGTGGCAAGAAGCTGACCCCGCGCCTTATCCGCCAGCTCGTCGACAAGGGCCTGAAG  240
N.galegae          GAATCCGGCAAAAAGCTCACGCCGCGCCTTCTCCGTCAGCTGACGGACAAGGGCCTGAAG  240
T4_1               GAATCCGGCAAGAAGCTCACCCCGCGTCTTCTCCGTCAGCTGACGGACAAGGGCCTCAAG  240
T4_8               GAATCCGGCAAGAAGCTCACCCCGCGTCTTCTCCGTCAGCTGACGGACAAGGGCCTCAAG  240
T22_47             GAATCCGGCAAGAAGCTCAACCCGCGTCTTCTCCGTCAGCTGACGGAGAAGGGCTTGAAG  240
T7_7               GAATCCGGCAAGAAGCTCAACCCGCGTCTTCTCCGTCAGCTGACGGAGAAGGGCTTGAAG  240
T5_2               GAATCCGGCAAGAAGCTCAACCCGCGTCTTCTCCGTCAGCTGACGGAGAAGGGCTTGAAG  240
T7_11              GAATCTGGCAAGAAGCTCAACCCGCGTCTTCTCCGTCAGCTGACGGAGAAGGGCTTGAAG  240
T6_21              GAATCCGGCAAGAAGCTCAACCCGCGTCTTCTCCGTCAGCTGACGGAGAAGGGCTTGAAG  240
T7_12              GAATCCGGCAAGAAGCTCAACCCGCGTCTTCTCCGTCAGCTGACGGAGAAGGGCTTGAAG  240
T16_9              GAATCCGGCAAGAAGCTCAACCCGCGTCTTCTCCGTCAGCTGACGGAGAAGGGCTTGAAG  240
T5_26              GAATCCGGCAAGAAGCTCAACCCGCGTCTTCTCCGTCAGCTGACGGAGAAGGGCTTGAAG  240
T24_19             GAATCCGGCAAGAAGCTCAACCCGCGTCTTCTCCGTCAGCTGACGGAGAAGGGCTTGAAG  240
T20_10             GAATCCGGCAAGAAGCTCAACCCGCGTCTTCTCCGTCAGCTGACGGAGAAGGGCTTGAAG  240
T11_12             GAATCCGGCAAGAAGCTCAACCCGCGTCTTCTCCGTCAGCTGACGGAGAAGGGCTTGAAG  240
T21_15             GAATCCGGCAAGAAGCTCAACCCGCGTCTTCTCCGTCAGCTGACGGAGAAGGGCTTGAAG  240
T25_7              GAATCCGGCAAGAAGCTCAACCCGCGTCTTCTCCGCCAGCTGACGGAGAAGGGCTTGAAG  240
T6_1               GAATCCGGCAAGAAGCTCAACCCGCGTCTTCTCCGTCAGCTGACGGAGAAGGGCTTGAAG  240
T13_2              GAATCCGGCAAGAAGCTCAACCCGCGTCTTCTCCGTCAGCTGACGGAGAAGGGCTTGAAG  240
T25_13             GAATCCGGCAAGAAGCTCAACCCGCGTCTTCTCCGTCAGCTGACGGAGAAGGGCTTGAAG  240
T23_12             GAATCCGGCAAGAAGCTCAACCCGCGTCTTCTCCGTCAGCTGACGGAGAAGGGCTTGAAG  240
T6_25              GAATCCGGCAAGAAGCTCAACCCGCGTCTTCTCCGTCAGCTGACGGAGAAGGGCTTGAAG  240
N.alkalisoli       GAGTCCGGCAAGAAGCTGACCCCGCGTCTGCTCCGGCAGCTGTCCGAGAAGGGCCTCAAG  240
N.huautlense       GAGTCGGGCAAGAAGCTGACCCCACGTCTGCTTCGGCAGCTGTCGGACAAGGGCCTCAAG  240
                   **    ***** ***** *  ** ** **  * ** *****    ** ****** * ***

A.tumefaciens      GCGCTTAAGGCGACTGACGAAGACCTCTACGGAAACTTCCTGGCCGAAGACATCGTCAAC  300
N.galegae          GCGTTGAAGGCAACCAATGACGACATCTACGGCAACTATCTTGCCGAAGACATCGTCAAC  300
T4_1               GCCCTGAAGGCCACCAATGACGACATCTACGGTAACTATCTTGCCGAAGACATCGTCAAC  300
T4_8               GCCCTGAAGGCCACCAATGACGACATCTACGGTAACTATCTTGCCGAAGACATCGTCAAC  300
T22_47             GCGTTGAAGGCGACCAATGACGACATCTACGGCAACTATCTTGCCGAAGACATCGTCAAC  300
T7_7               GCGTTGAAGGCGACCAATGACGACATCTACGGCAACTATCTTGCCGAAGACATCGTCAAC  300
T5_2               GCGTTGAAGGCGACCAATGACGACATCTACGGCAACTATCTTGCCGAAGACATCGTTAAC  300
T7_11              GCGTTGAAGGCGACCAATGACGACATCTACGGCAACTATCTTGCCGAAGACATCGTCAAC  300
T6_21              GCGTTGAAGGCGACCAATGACGACATCTACGGCAACTATCTTGCCGAAGACATCGTCAAC  300
T7_12              GCGTTGAAGGCGACCAATGACGACATCTACGGCAACTATCTTGCCGAAGACATCGTCAAC  300
T16_9              GCGTTGAAGGCAACCAATGACGACATCTACGGCAACTATCTTGCCGAAGACATCGTCAAC  300
T5_26              GCCTTGAAGGCGACCAATGACGACATCTACGGCAACTATCTTGCCGAAGACATCGTCAAC  300
T24_19             GCCTTGAAGGCGACCAATGACGACATCTACGGCAACTATCTTGCCGAAGACATCGTCAAC  300
T20_10             GCCTTGAAGGCGACCAATGACGACATCTACGGCAACTATCTTGCCGAAGACATCGTCAAC  300
T11_12             GCCTTGAAGGCGACCAATGACGACATCTACGGCAACTATCTTGCCGAAGACATCGTCAAC  300
T21_15             GCCTTGAAGGCGACCAATGACGACATCTACGGCAACTATCTTGCCGAAGACATCGTCAAC  300
T25_7              GCCTTGAAGGCGACCAATGACGACATCTACGGCAACTATCTTGCCGAAGACATCGTCAAC  300
T6_1               GCCTTGAAGGCGACCAATGACGACATCTACGGCAACTATCTTGCCGAAGACATCGTCAAC  300
T13_2              GCGTTGAAGGCAACCAATGACGACATCTACGGCAACTATCTTGCCGAAGACATCGTCAAC  300
T25_13             GCCTTGAAGGCGACCAATGACGACATCTACGGCAATTATCTTGCCGAAGACATCGTCAAC  300
T23_12             GCCTTGAAGGCGACCAATGACGACATCTACGGCAACTATCTTGCCGAAGACATCGTCAAC  300
T6_25              GCCCTGAAGGCGACCAATGACGACATCTACGGCAACTATCTTGCCGAAGACATCGTCAAC  300
N.alkalisoli       GCACTCAAGGCAACCAACGACGATCTCTACGGCAACTATCTCGCCGAAGATGTCGTCAAC  300
N.huautlense       GCGCTCAAGGCAACCAATGACGATCTCTACGGCAACTATCTCGCCGAAGACATCGTCAAC  300
                   **  * ***** **  * ** **  ******* ** *  ** ********  **** ***

A.tumefaciens      TACGAGACGGGTGAAATCTATCTCGAAGCTGGCGACGAAATCGACGAGAAGACGCT      356
N.galegae          GCGGCAACCGGCGAGATCTACCTCGAAGCCGGCGACGAGATCGACGAAAAGACCCT      356
T4_1               GCGGCGACCGGCGAAATCTACCTCGAAGCCGGCGACGAGATCGACGAAAAGACCCT      356
T4_8               GCGGCGACCGGCGAAATCTACCTCGAAGCCGGCGACGAGATCGACGAAAAGACCCT      356
T22_47             GCGGCAACCGGCGAGATCTACCTCGAAGCCGGCGACGAAATCGACGAAAAGACCCT      356
T7_7               GCGGCAACCGGCGAGATCTACCTCGAAGCCGGCGACGAAATCGACGAAAAGACCCT      356
T5_2               GCGGCAACCGGCGAGATCTACCTCGAAGCCGGCGACGAAATCGACGAAAAGACCCT      356
T7_11              GCGGCAACCGGCGAGATCTACCTCGAAGCCGGCGACGAAATCGACGAAAAGACCCT      356
T6_21              GCGGCAACCGGCGAGATCTACCTCGAAGCCGGCGACGAAATCGACGAAAAGACCCT      356
T7_12              GCGGAAACCGGCGAGATCTACCTCGAAGCCGGCGACGAAATCGACGAAAAGACCCT      356
T16_9              GCGGCAACCGGCGAGATCTACCTCGAAGCCGGCGACGAAATCGACGAAAAGACCCT      356
T5_26              GCGGCAACCGGCGAGATCTACCTCGAAGCCGGCGACGAAATCGACGAAAAGACCCT      356
T24_19             GCGGCAACCGGCGAGATCTATCTCGAAGCCGGCGACGAAATCGACGAAAAGACCCT      356
T20_10             GCGGCAACCGGCGAGATCTACCTCGAAGCCGGCGACGAAATCGACGAAAAGACCCT      356
T11_12             GCGGCAACCGGCGAGATCTATCTCGAAGCCGGCGACGAAATCGACGAAAAGACCCT      356
T21_15             GCGGCAACCGGCGAGATCTATCTCGAAGCCGGCGACGAAATCGACGAAAAGACCCT      356
T25_7              GCGGCAACCGGCGAGATCTACCTCGAAGCCGGCGACGAAATCGACGAAAAGACCCT      356
T6_1               GCGGCAACCGGCGAAATCTACCTCGAAGCCGGCGACGAAATCGACGAAAAGACCCT      356
T13_2              GCGGCAACCGGCGAGATCTACCTCGAAGCCGGCGACGAAATCGACGAAAAGACCCT      356
T25_13             GCGGCAACCGGCGAGATCTACCTCGAAGCCGGCGACGAAATCGACGAAAAGACCCT      356
T23_12             GCGGCAACCGGCGAGATCTACCTCGAAGCCGGCGACGAAATCGACGAAAAGACCCT      356
```

```
T6_25            GCGGCAACCGGCGAGATCTACCTCGAAGCCGGCGACGAAATCGACGAAAAGACCCT      356
N.alkalisoli     ACGGAAACCGGTGAGATTTATCTCGAGGCCGGCGACGAGATCGACGAGAAGACCCT      356
N.huautlense     ATGGAAACCGGTGAGATTTATCTCGAAGCCGGCGACGAGATCGACGAGAAGACCCT      356
                    *  ** ** ** ** ** ***** ** ******** ******** ***** **
```
