# Supplementary material for: Novel, non-symbiotic isolates of Neorhizobium from a dryland agricultural soil
Source: PeerJ. 2018 May 16;6:e4776. doi: 10.7717/peerj.4776 (PMC5960266; doi:10.7717/peerj.4776)
Supplement: Table S1 [file peerj-06-4776-s003.docx]

Physicochemical properties of Tomejil soil

| Property |  | Tomejil soil |
| --- | --- | --- |
| (pH) (Ext.1:2.5)^1^ |  | 8.1 |
| Pretest salinity^1^ (µS/cm) (Ext.1:5) |  | 239 |
|  |  |  |
| Organic matter (%)^1^ |  | 2.48 |
| Total nitrogen (%)^1^ |  | 0.192 |
| Carbonates  (% CaCO_3_)^2^ |  | 34.5 |
| Exchangeable cation (ppm)^1^ | Na | 80 |
|  | K | 2,279 |
|  | Ca | 7,138 |
|  | Mg | 247 |
|  | P (Olsen) | 40.7 |
|  |  |  |
| Texture  (Bouyoucos method)^1^ | Clay | 17.2 |
|  | Silt | 38.2 |
|  | Sand | 44.6 |
