# Supplementary material for: Novel, non-symbiotic isolates of Neorhizobium from a dryland agricultural soil
Source: PeerJ. 2018 May 16;6:e4776. doi: 10.7717/peerj.4776 (PMC5960266; doi:10.7717/peerj.4776)
Supplement: Table S2 [file peerj-06-4776-s004.docx]

GenBank IDs of DNA sequences obtained or used in this work.

|  | GenBank ID | | |
| --- | --- | --- | --- |
| Strain | 16S rDNA | *rpoB* | Genome |
| *Neorhizobium* sp**.** T9_24 | MG957216 | MG966473 |  |
| *Neorhizobium* sp. T6_25 | MG957217 | MG966474 | PRJNA434367 |
| *Neorhizobium* sp. T13_2 | MG957218 | MG966475 |  |
| *Neorhizobium* sp. T7_12 | MG957219 | MG966476 | PRJNA434366 |
| *Neorhizobium* sp. T6_21 | MG957220 | MG966477 |  |
| *Neorhizobium* sp. T6_1 | MG957221 | MG966478 |  |
| *Neorhizobium* sp. T20_22 | MG957222 | MG966479 | PRJNA434360 |
| *Neorhizobium* sp. T25_27 | MG957223 | MG966480 | PRJNA434365 |
| *Neorhizobium* sp. T4_8 | MG957224 | MG966481 |  |
| *Neorhizobium* sp. T4_1 | MG957225 | MG966482 |  |
| *Neorhizobium* sp. T25_7 | MG957226 | MG966483 |  |
| *Neorhizobium* sp. T25_13 | MG957227 | MG966484 | PRJNA434364 |
| *Neorhizobium* sp. T21_15 | MG957228 | MG966485 |  |
| *Neorhizobium* sp. T24_19 | MG957229 | MG966486 |  |
| *Neorhizobium* sp. T7_7 | MG957230 | MG966487 |  |
| *Neorhizobium* sp. T16_9 | MG957231 | MG966488 |  |
| *Neorhizobium* sp. T5_2 | MG957232 | MG966489 |  |
| *Neorhizobium* sp. T7_11 | MG957233 | MG966490 |  |
| *Neorhizobium* sp. T22_47 | MG957234 | MG966491 |  |
| *Neorhizobium* sp. T23_12 | MG957235 | MG966492 |  |
| *Neorhizobium galegae* HAMBI 540^T^ |  |  | PRJNA224116 |
| *Neorhizobium alkalisoli* CCBAU 01393^T^ | EU074168.1 | KF278573.1 |  |
| *Neorhizobium huautlense* LMH 18254^T^ | AF025852.1 | EF217309.1 |  |
| *Agrobacterium tumefaciens* NCPPB 2437^T^ | NR_115516.1 | JN580757.1 |  |
| *Neorhizobium alkalisol*i DSM 21817^T^ |  |  | PRJNA434370 |
| *Neorhizobium huautlense* DSM 21826^T^ |  |  | PRJNA434371 |
| *Agrobacterium tumefaciens* Ach5 |  |  | PRJNA278497 |
