# Supplementary material for: Novel, non-symbiotic isolates of Neorhizobium from a dryland agricultural soil
Source: PeerJ. 2018 May 16;6:e4776. doi: 10.7717/peerj.4776 (PMC5960266; doi:10.7717/peerj.4776)
Supplement: Table S3 — Genome sequences similar to the conjugal transfer genes from Agrobacterium tumefaciens Ach5 Ti plasmid (RefSeq: NZ_CP007228) were searched for in genome sequences by BLAST. [file peerj-06-4776-s005.docx]

Presence of plasmid conjugal transfer genes in the genomes of Tomejil strains.

Genome sequences similar to the conjugal transfer genes from *Agrobacterium tumefaciens* Ach5 Ti plasmid (RefSeq: NZ_CP007228) were searched for in genome sequences by BLAST.

| Genome | *tra* genes^a^ | *trb* genes^a^ |
| --- | --- | --- |
| T20_22 | – | – |
| T7_12 | – | *trb LGI* |
| T25_27 | – | – |
| T25_13 | + | + |
| T6_25 | – | – |

^a^ – : Absent; + : A complete gene set present; *trb LGI* : only these genes were present.
