# Supplementary material for: Novel, non-symbiotic isolates of Neorhizobium from a dryland agricultural soil
Source: PeerJ. 2018 May 16;6:e4776. doi: 10.7717/peerj.4776 (PMC5960266; doi:10.7717/peerj.4776)
Supplement: Table S4 [file peerj-06-4776-s006.docx]

Pairwise Average Nucleotide Identities (ANIb) between representative *Neorhizobium* sp. Tomejil isolates and *Neorhyzobium* sp. type strains

|  | *N. galegae* HAMBI 540^T^ | *N. alkalisoli* DSM 21826^T^ | *N. huautlense* DSM 21817^T^ | T7_12 | T25_27 | T25_13 | T6_25 | T20_22 |
| --- | --- | --- | --- | --- | --- | --- | --- | --- |
| *N. galegae* HAMBI 540^T^ | * | 84.75 | 84.51 | 91.70 | 91.70 | 91.76 | 91.59 | 91.71 |
| *N. alkalisoli* DSM 21826^T^ | 84.74 | * | 89.40 | 84.77 | 84.83 | 84.79 | 84.75 | 84.76 |
| *N. huautlense* DSM 21817^T^ | 84.52 | 89.41 | * | 84.61 | 84.66 | 84.61 | 84.50 | 84.59 |
| T7_12 | 91.70 | 84.77 | 84.61 | * | 97.43 | 96.47 | 95.51 | 97.43 |
| T25_27 | 91.69 | 84.83 | 84.66 | 97.43 | * | 96.45 | 95.53 | 97.73 |
| T25_13 | 91.76 | 84.79 | 84.61 | 96.47 | 96.45 | * | 95.50 | 96.46 |
| T6_25 | 91.59 | 84.76 | 84.51 | 95.51 | 95.53 | 95.50 | * | 95.51 |
| T20_22 | 91.71 | 84.76 | 84.59 | 97.43 | 97.73 | 96.46 | 95.51 | * |
